# The Association between Gut Microbiota and Serum Biomarkers in Children with Atopic Dermatitis

**DOI:** 10.3390/biomedicines12102351

**Published:** 2024-10-15

**Authors:** Irina G. Kalashnikova, Alexandra I. Nekrasova, Anna V. Korobeynikova, Maria M. Bobrova, German A. Ashniev, Sirozhdin Yu. Bakoev, Angelica V. Zagainova, Mariya V. Lukashina, Larisa R. Tolkacheva, Ekaterina S. Petryaikina, Alexander S. Nekrasov, Sergey I. Mitrofanov, Tatyana A. Shpakova, Lidiya V. Frolova, Natalya V. Bulanova, Ekaterina A. Snigir, Vladimir E. Mukhin, Vladimir S. Yudin, Valentin V. Makarov, Anton A. Keskinov, Sergey M. Yudin

**Affiliations:** Federal State Budgetary Institution “Centre for Strategic Planning and Management of Biomedical Health Risks” of the Federal Medical and Biological Agency, Pogodinskaya Str., 10/1, 119121 Moscow, Russia; akinshina@cspfmba.ru (A.I.N.); akorobeinikova@cspfmba.ru (A.V.K.); mbobrova@cspfmba.ru (M.M.B.); gashniev@cspfmba.ru (G.A.A.); sbakoev@cspfmba.ru (S.Y.B.); azagaynova@cspmz.ru (A.V.Z.); mlukashina@cspmz.ru (M.V.L.); ltolkacheva@cspfmba.ru (L.R.T.); epetryakina@cspfmba.ru (E.S.P.); anekrasov@cspfmba.ru (A.S.N.); mitrofanov@cspfmba.ru (S.I.M.); maslova@cspfmba.ru (T.A.S.); lfrolova@cspfmba.ru (L.V.F.); nbulanova@cspfmba.ru (N.V.B.); esnigir@cspfmba.ru (E.A.S.); vmukhin@cspfmba.ru (V.E.M.); vyudin@cspfmba.ru (V.S.Y.); makarov@cspfmba.ru (V.V.M.); keskinov@cspfmba.ru (A.A.K.); yudin@cspmz.ru (S.M.Y.)

**Keywords:** gut microbiota, microbiome, atopic dermatitis, atopy, dysbiosis, 16S rRNA, cytokines, multiplex analysis, correlation analysis

## Abstract

**Background.** Currently, it is known that the gut microbiota plays an important role in the functioning of the immune system, and a rebalancing of the bacterial community can arouse complex immune reactions and lead to immune-mediated responses in an organism, in particular, the development of atopic dermatitis (AD). Cytokines and chemokines are regulators of the innate and adaptive immune response and represent the most important biomarkers of the immune system. It is known that changes in cytokine profiles are a hallmark of many diseases, including atopy. However, it remains unclear how the bacterial imbalance disrupts the function of the immune response in AD. **Objectives**. We attempted to determine the role of gut bacteria in modulating cytokine pathways and their role in atopic inflammation. **Methods.** We sequenced the 16S rRNA gene from 50 stool samples of children aged 3–12 years who had confirmed atopic dermatitis, and 50 samples from healthy children to serve as a control group. To evaluate the immune status, we conducted a multiplex immunofluorescence assay and measured the levels of 41 cytokines and chemokines in the serum of all participants. **Results.** To find out whether changes in the composition of the gut microbiota were significantly associated with changes in the level of inflammatory cytokines, a correlation was calculated between each pair of bacterial family and cytokine. In the AD group, 191 correlations were significant (Spearman’s correlation coefficient, *p* ≤ 0.05), 85 of which were positive and 106 which were negative. **Conclusions**. It has been demonstrated that intestinal dysbiosis is associated with alterations in cytokine profiles, specifically an increase in proinflammatory cytokine concentrations. This may indicate a systemic impact of these conditions, leading to an imbalance in the immune system’s response to the Th2 type. As a result, atopic conditions may develop. Additionally, a correlation between known AD biomarkers (IL-5, IL-8, IL-13, CCL22, IFN-γ, TNF-α) and alterations in the abundance of bacterial families (*Pasteurellaceae, Barnesiellaceae, Eubacteriaceae*) was observed.

## 1. Introduction

The gut microbiome contains, according to various estimates, between 10^13^ and 10^14^ microorganisms, which are in a symbiotic relationship with each other and the human body [1]. The composition of the human microbiota is unique to each individual and depends on a variety of factors, both endogenous and exogenous. It begins to form at the moment of birth and continues to evolve throughout life [2]. Intestinal bacteria are involved in many organism processes, and intestinal dysbiosis can lead to the development of various conditions [3]. Due to the metabolic potential, the intestinal microbiota affects not only the intestinal mucosa but also the entire organism as a whole, which makes it possible to consider the microbiota as an important factor in the regulation of the immune system [4].

Atopic dermatitis (AD) is a complex disease that has various aspects to its pathogenesis. One of these aspects is the dysbiosis of the intestinal microbiota, which has been linked to the development of AD [5,6,7]. Known biomarkers of atopic dermatitis include IL-22 (Interleukin-22), IL-18, and IL-13, as well as eosinophilia and elevated IgE levels. Chemokines that correlate with disease severity, such as eotaxin/CCL11 (eosinophil chemotactic protein/C-C motif chemokine 11), MDC/CCL22 (macrophage-derived chemokine), and TARC/CCL17 (thymus and activation-regulated chemokine), are also associated with the condition [8,9]. It is assumed that intestinal dysbiosis may cause the activation of Th2 cells that produce IL-4, IL-5, and IL-13, contributing to the development of inflammation. IL-4 and IL-13 stimulate B cells to produce IgE and also promote the eosinophilic reaction, which is associated with atopic dermatitis and other accompanied atopic diseases [10,11,12]. Normally, exposure to a healthy gut microbiota stimulates the development of Th1 cells and helps to modulate immune tolerance, supporting the Th1/Th2 balance [13]. Some researchers suggest that one possible cause of the development of atopic conditions may also be a disruption of the mechanisms that form immune tolerance during early childhood [2,10]. The development of the microbiome during early life occurs concurrently with the development of the immune system. This is certainly important for the pathogenesis of many diseases [14,15]. A healthy balance between the developing immune system and gut microbiota is crucial to prevent the future development of immune-related diseases [16].

Cytokines and chemokines are important signaling molecules produced by immune cells that modulate multiple processes in the organism [17]. It is known that changes in cytokine profiles are observed in various autoimmune conditions, including atopy [9,18]. However, it remains unclear how bacterial imbalance disrupts the function of the immune response in atopic dermatitis. For example, a correlation between certain serum cytokines and bacterial taxa in the gut microbiome has been previously noted in other conditions [19,20,21]. In addition, the immunomodulatory effect of certain bacterial taxa has been shown [22], and changes in cytokine profiles were one of the signs of this relationship. It was noted that the most microbiota-dependent immune markers are IL-1α, IL-1β, IL-6, IL-22, TNF-α (tumor necrosis factor alpha), IL-12, and IFN-γ (interferon gamma) [22]. Unfortunately, due to the wide variety of experimental procedures and research designs, it is difficult to clearly identify specific signs of atopy in the intestinal microbiota of children with atopic dermatitis. This is a common challenge for researchers in various fields [23,24]. Nevertheless, several researchers have identified common bacterial patterns associated with atopic dermatitis. These include a decrease in microbial diversity, a reduction in the number of short-chain fatty acid producers, and an increase in the potentially pathogenic representatives of the gut microbiota. Despite the fact that the prevalence of certain taxa may differ depending on various factors, a general trend toward the development of a proinflammatory atopic phenotype is observed in most patients [5,25,26].

Therefore, in our study, we analyzed the relationship between changes in cytokine/chemokine levels characteristic of atopy in children and the state of the gut microbiome. However, the detailed mechanisms of the cross-interaction of certain bacterial taxa of the gut microbiota and the host immune system are the subject of further research. Understanding how the gut microbiota affects immune phenotypes will help further determine the degree of human susceptibility to certain diseases, as well as the search for new therapeutic approaches.

## 2. Materials and Methods

### 2.1. Study Design and Population

#### 2.1.1. Participants

The main group consisted of 50 participants aged 3 to 12 years with an IgE-associated diagnosis of atopic dermatitis. The control group included 50 healthy children. Both groups were comparable in terms of gender and age. The study was conducted over a three-month period from September to December 2020.

The study included patients who met the following criteria.

In the group of healthy children (control):

- Age between 3 and 12 years;
- Absence of atopic dermatitis and food allergies;
- Absence of infectious diseases in the acute phase;
- Absence of chronic diseases of the gastrointestinal tract, cardiovascular system, kidneys, respiratory organs, or endocrine disorders;
- No regular intake of medicines, or biologically active supplements (including probiotics), or antibiotics (including other antibacterial drugs), or probiotics in the last two months;
- Availability of a set of necessary documents signed by a parent or legal representative of the child;
- Sampling of stool and blood samples that do not affect the patient’s current health status.

In the group of children with atopic dermatitis (AD):

- A confirmed diagnosis of atopic dermatitis, with elevated levels of immunoglobulin E, and possible food allergies (but these are not exclusion criteria);
- Availability of necessary documents signed by a parent or legal representative of the child;
- Collection of stool and blood samples that do not affect the patient’s current health;
- Age between 3 and 12 years;
- Absence of any acute or chronic infectious diseases, including those affecting the gastrointestinal, cardiovascular, respiratory, renal, endocrine or oncological systems. Also, autoimmune diseases are not allowed;
- No current medications or supplements, including probiotics, or antibiotics, or probiotics use in the last two months.

#### 2.1.2. Ethical Statements

The study was conducted in accordance with the guidelines of the Helsinki Declaration and approved by the Committee on Institutional Ethics of the Federal State Budgetary Scientific Institution “Federal Research Centre of Nutrition, Biotechnology and Food Safety”, Moscow, Russia (Protocol No. 5, dated 2 July 2020). The Set of documents signed by the parent or legal guardian of the child included:

1. Informed consent to participate in the research study. The informed consent form is available in the Supplementary Materials. We used the informed consent form, as we did in our previous study [27].
2. Informed consent regarding the processing of personal information.
3. Informed permission for medical procedures.

A signed set of documents and written informed consent to participate were received from the legal representatives of all study participants.

Each participant in the study was given a unique identifier based on their registration information at the beginning of the study. This identifier was used throughout the study to ensure anonymity and protect the privacy of participants’ personal information.

#### 2.1.3. Samples

After obtaining the informed consent from the legal representative of each participant, all participants underwent a medical history collection, a physical examination, and the collection of blood serum and stool samples. All samples were obtained once during the initial visit of the participant following their enrollment in the study, in accordance with the inclusion criteria. Prior to the start of the study, all participants were instructed not to take antibiotics, probiotics, prebiotics, or laxatives for at least one month. After collecting the biological samples, they were transported to the laboratory within one to three hours and then stored at −80 °C until analysis.

### 2.2. DNA Isolation and Library Preparation

Amplicon sequencing of the marker variable region V3–V4 of bacterial 16S rRNA genes was used to study the composition of the microbiota. Total bacterial DNA from stool samples was extracted using a QIAamp DNA Stool Mini Kit (Qiagen, Hilden, Germany) according to the manufacturer’s instructions. Amplicon libraries of 16S rRNA were prepared using PCR with universal primers for the V3-V4 region. A set of MiSeq V2 Nano reagents was used to prepare DNA for sequencing. Sequencing was performed on a MiSeq device (Illumina, San Diego, CA, USA), as a result of which reads with a length of 150 bp were obtained from each end of the amplicon. The amount of PhiX Control v3 was at least 1%.

### 2.3. Multiplex Analysis of the Cytokine/Chemokine Profiles of Blood Serum

The analysis was performed on the blood serum of the participants using K2-EDTA. The concentration of cytokines in blood serum was determined by a FLEXMAP 3D device (Luminex, Austin, TX, USA) using Human Cytokine/Chemokine Magnetic Bead Panel Cat reagent kits, #HCYTOMAG-60K (EMD Millipore Corporation, Burlington, MA, USA). All measurement steps were carried out in accordance with the manufacturer’s protocol. The analysis of the obtained data was carried out using the standard MILLIPLEX^®^ Analyst 5.1 program. Each sample was analyzed in two repeats, and the average value for the two measurements was used as the measured concentration. The list of analytes is presented in Table S1.

### 2.4. Processing and Analysis of Metagenomic Data

The Quantitative Insights in Microbial Ecology 2 (QIIME2) software, version 2022.2.0, was employed to process and analyze the sequencing data [28]. The cutadapt plugin, part of the QIIME2 toolkit, was used to remove primer residues in the V3-V4 flanking areas that could potentially contaminate the data post-automatic processing [29]. The DADA2 tool [30] was then utilized to trim, filter, and merge the reads into contigs, which resulted in a table containing all the amplicon sequence variants (ASVs) identified in each sample, along with their actual and relative representation. Subsequently, a phylogenetic tree was constructed using the FastTree plugin [31], with the root at the midpoint. This process involved a multiple sequence alignment using MAFFT [32]. The taxonomy of the ASVs was assigned using the Naive Bayes classifier from the scikit-learn library. This classifier was trained on 16S rRNA reference databases, specifically SILVA (v.138.99) and RDP (v.11.5). The resulting tables were then merged with the ASV data. The final stage involved a detailed analysis of the taxonomy at the family level.

### 2.5. Statistical Analysis

#### 2.5.1. Software

The analysis was performed using the R programming language (version 4.2.1) with average data processing by R Studio 2022.02.3 (build 492).

#### 2.5.2. Data Preprocessing

The phyloseq format (phyloseq v. 1.16.2 package) was used for calculations, which converts data into a specialized S4 class system format and ensures data connectivity and consistency during operation. The analysis was carried out at the level of bacterial families, and the tax_glom() function was used to combine taxa by their taxonomic affiliation. After the agglomeration procedure, user data normalization was performed in order to minimize the effects of small taxa: all values in the ASV frequency table below 31 were replaced by 0; the values from 31 to 99 were replaced by 75, and those from 100 to 149 by 125. Values outside these ranges remained unchanged. All completely null families were removed from the analysis.

#### 2.5.3. Alpha and Beta Diversity Analysis

Alpha diversity refers to the diversity within a sample and is measured as the number of species (richness) and the evenness of species abundances. In the context of gut microbiota, alpha diversity provides insights into the complexity and variety of microbial communities within a single sample or individual. High alpha diversity is often associated with greater ecosystem stability and resilience, and it is generally considered beneficial for health. Beta diversity, on the other hand, measures the diversity between different individuals or sample groups, providing a comparative analysis of microbial community composition. It helps in identifying patterns of microbial distribution and the factors driving these differences, such as diet, environment, or disease states.

During the alpha diversity analysis, four alpha diversity indices were considered: Shannon, Pielou, Simpson, and Strong. The nonparametric statistical Mann–Whitney test with FDR refinement (rstatix v.0.7.2) was used to compare the values. The results are visualized as distribution graphs using the ggplot2 v.3.5.0 package.

To assess the beta diversity of the community, weighted and unweighted UniFrac distance matrices were calculated using the UniFrac() function of the phyloseq package. The results are visualized in three-dimensional space using the plotly package (v.4.10.4). Based on the distance matrix, a PERMANOVA variance analysis was performed (vegan v.2.6-4 package).

#### 2.5.4. Statistical Analysis of the Population

In order to identify the families that make the greatest contribution to the difference between the control group and AD, two microbiome analysis tools were used: LEfSe (effect size of linear discriminant analysis, microbiomeMarker v.1.4.0) and Coda4microbiome (penalty regression, coda4microbiome v.0.2.3).

Differences in cytokine levels were evaluated similarly to the alpha diversity analysis item: statistical reliability was checked using the Mann–Whitney criterion (with clarification by FDR), and all cytokines that passed the significance threshold (α = 0.05) were visualized to graphically illustrate differences in their concentrations.

Multidimensional NMDS analysis was used to study the effect of cytokines on the structure of the bacterial community (vegan v.2.6-4 package). This technique uses an arbitrary distance matrix and creates a two-dimensional projection of the mutual distances between the objects of study. At the first stage of the work, the best distance metric is determined (by calculating the Spearman correlation between two matrices), and the rankindex() function is used. Next, the metaMDS() function performs non-metric scaling, and the envfit() function calculates the correlation of indicators with the ordination axes and gives an estimate of statistical significance based on a permutation test. To search for bacterial families with which the identified cytokines in atopic dermatitis have relationships, an analysis was performed using a generalized linear model (stats package v.4.2.3).

#### 2.5.5. Correlation Analysis

Based on the results of the previous stage, a search was carried out for the relationship between the level of significant cytokines and alpha diversity. Correlations and their statistical significance were considered using the ggscatter() function of the ggpubr package (v.0.6.0.999).

Further, a correlation analysis was performed between the level of cytokines and the relative number of bacteria within each group. Spearman’s method was used for the calculation, an estimate of the statistical significance (*p*-value) of each correlation pair was obtained, and in order to avoid false positive results, the value was adjusted using the FDR method. To detail and visualize the interrelationships of circulating cytokine/chemokine concentrations with the individual bacterial taxa of the intestinal microbiota of children with AD, heat maps were visualized indicating statistical significance (microbiomeSeq v.0.1).

## 3. Results

### 3.1. Diversity Analysis of the Gut Microbiota

**Figure 1:**
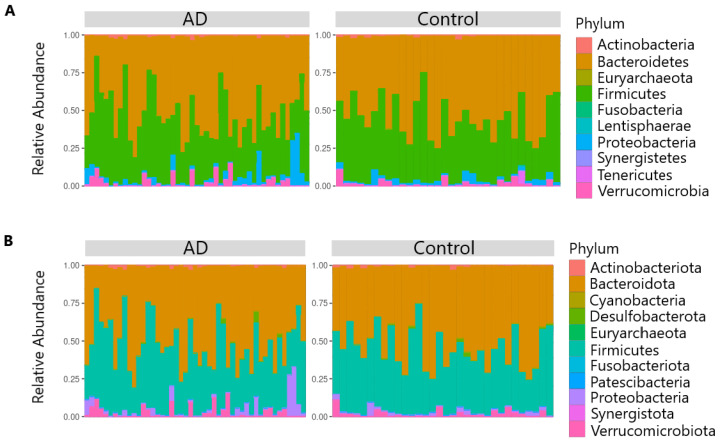
The relative abundance of bacteria at the type level between the groups in the RDP (**A**) and SILVA (**B**) data.

To check the quality of the samples, a measure of the sequencing depth was used, the coverage threshold was indicated at 20 thousand, and as a result, 81 samples exceeded the threshold (49 AD, 32 control). Before the analysis, the Rosner criterion was also applied to search for an outlier in the data, according to the results of which 47 samples with AD and 32 control cases were included in the analysis. Using the transform_sample_counts() function, the relative number of bacteria was obtained, after which histograms by groups were constructed using the plot_bar() function, which show the ratios of the number of bacterial types for each sample in the analysis (Figure 1).

**Figure 2:**
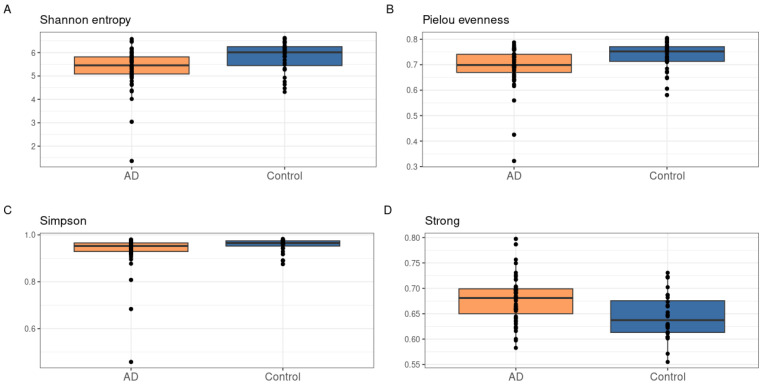
The distribution of alpha diversity index values between groups, with indications of statistical significance ((**A**)—Shannon entropy, (**B**)—Pielou evenness index, (**C**)—Simpson index, (**D**)—Strong index). The alpha diversity indices differ significantly between groups (α = 0.05).

According to Figure 1, the most prevalent types are *Bacteroidetes*, *Firmicutes*, *Proteobacteria*, and *Verrucomicrobia*. Patients with atopy have a higher proportion of *Firmicutes* and *Proteobacteria* compared to the control group. Next, we analyzed the diversity and consistency of bacterial communities in both groups and the differences between them. We found significant differences in alpha diversity using four indices, Shannon, Pielou, Simpson, and Strong (*p*-value < 0.05), as shown in Figure 2.

The statistical significance of the differences between the groups of atopic dermatitis and control was assessed using the Mann–Whitney criterion with the correction of multiple comparison of FDR. As can be seen from Figure 2, the Strong index is higher in patients with AD, which indicates that the relative unevenness of the bacterial community diversity is higher in this group.

Weighted and unweighted UniFrac distance matrices were used to assess the beta diversity of microbial communities. The results were visualized in three-dimensional space (Figure S1). To determine whether there are differences in the microbial communities depending on the presence or absence of the disease, we conducted a permutation analysis of variance, the results of which are presented in Table 1. The coefficient F is the ratio between two standard values. If the null hypothesis about the equality of the groups is correct, the F-value should be approximately one. A higher F-value indicates a greater difference between the means of the groups. Patients with AD have a different composition of intestinal microbiota compared to healthy individuals. This is characterized by a lower richness and diversity in their gut microbiota.

### 3.2. Changes in the Composition of the Microbiota Associated with Atopic Dermatitis

**Figure 3:**
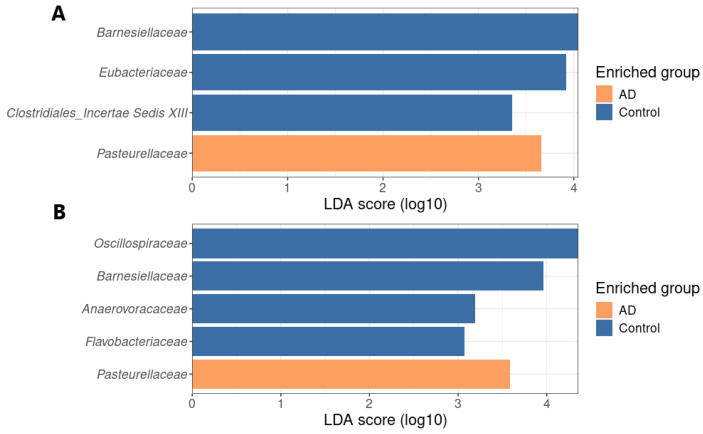
Taxonomic differences of the fecal microbiota in AD relative to the control group. LEfSe results for RDP (**A**) and SILVA (**B**) data; histogram with identified biomarkers indicating enrichment and representation score (LDA score). Cladograms with bacterial families with significant differences in groups are shown in Figure S2.

The comparison of the group of participants with AD relative to the control group was carried out using linear discriminant analysis (LDA) effect size LEfSe (Figure 3 and Figure S2) and the coda4microbiome algorithm based on logarithmic coefficients (penalty regression model) [33].

**Figure 4:**
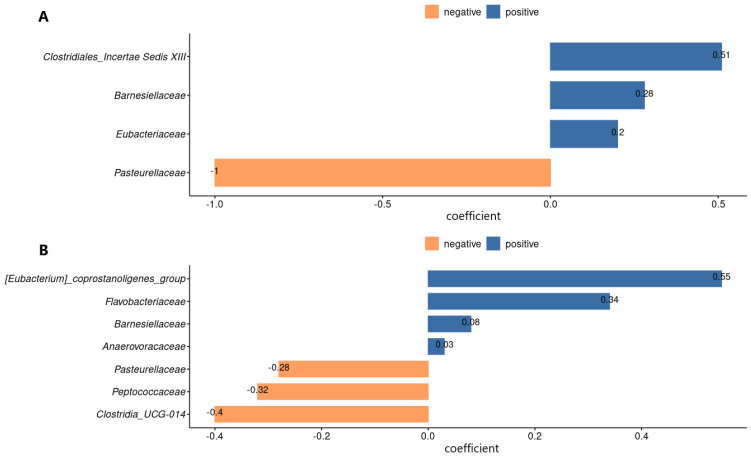
Results of the RDP (**A**) and SILVA (**B**) penalty regression models: taxa that best distinguish between patients with AD and the control group. The magnitude of the coefficients indicates the contribution of each variable to the model.

According to the results of the LEfSe analysis, four significant biomarkers were identified when analyzing the RDP data: *Barnesiellaceae*, *Eubacteriaceae*, *Clostridiales Incertae Sedis XIII*—enrichment in the control group, *Pasteurellaceae*—enrichment in AD group. In the case of SILVA data, five significant bacterial families were found: *Oscillospiraceae*, *Barnesiellaceae*, *Anaerovoraceae*, *Flavobacteriaceae*—enrichment in the control group, *Pasteurellaceae*—enrichment in the AD group. The coda4microbiome research tool was used to identify taxa closely related to the status of the disease. Calculations are based on logarithmic coefficients: the algorithm examines the relationship of each paired logarithmic ratio with a dependent variable. The results are shown in Figure 4.

Bacteria with a negative coefficient are represented in the AD group, while positive coefficient values indicate enrichment in the control group. The analysis of penalty regression and linear discriminant analysis revealed the same biomarkers in the analysis of RDP; however, in the case of SILVA, the families of *Peptococcaceae* and *Clostridia UCG-014* with enrichment in the AD group and *Eubacterium coprostanoligenes group* with enrichment in the control group were additionally detected during penalty regression. The *Oscillospiraceae* family has not been identified.

### 3.3. Results of Cytokine Level Analysis

**Figure 5:**
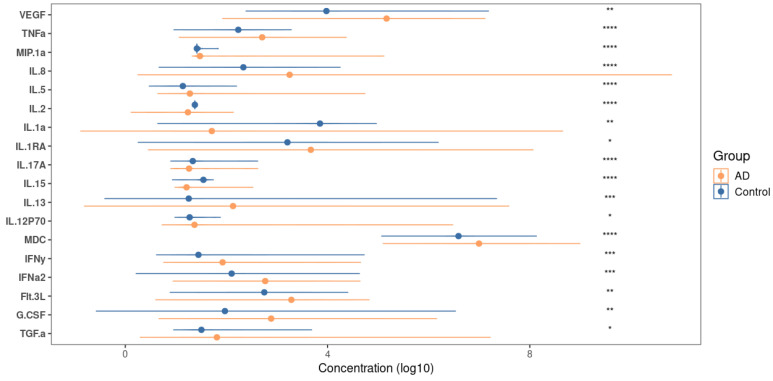
Comparison of the concentrations of individual cytokines in the serum of participants with AD and the control group; significance assessment indicated on the right side of the graph. Significance is denoted as follows: <0.0001: ‘****’, <0.001: ‘***’, <0.01: ‘**’, <0.05: ‘*’.

In order to determine the state of immunity, we evaluated the cytokine profiles of the participants, where we compared the results of the levels of individual markers in patients with atopic dermatitis with the control group (Figure 5).

Thus, a significant increase in the levels (*p* < 0.05) of several markers that are characteristic of the Th2 response and atopy, such as MDC/CCL22 (macrophage-derived chemokine/C-C motif chemokine 22), IL-5, IL-8, IL-13, IFN-γ, TNF-α, MIP-1α/CCL3 (macrophage inflammatory protein 1-α/chemokine (C-C motif) ligand 3), and VEGF (vascular endothelial growth factor), was observed in the group of participants with atopy. Additionally, there was a decrease in the levels of Th1-type cytokines, such as IL-2 and IL-15.

### 3.4. Association of Changes in Cytokine Profile and Microbiome Composition

**Figure 6:**
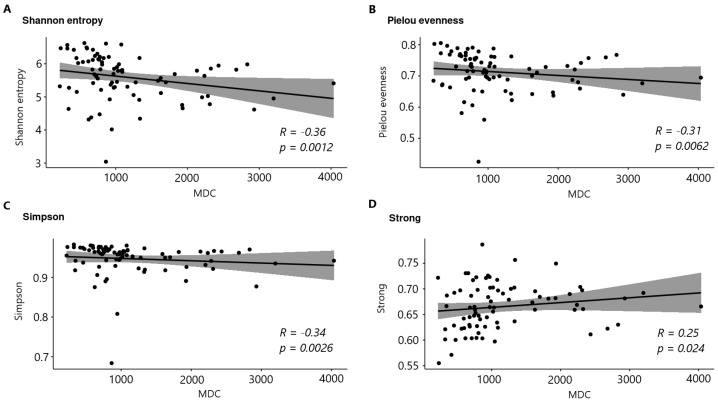
Correlations of the MDC chemokine and alpha diversity indices ((**A**) Shannon entropy, (**B**) Pielu evenness index, (**C**) Simpson index, (**D**) Strong index).

The study analyzed the interrelationships between the indices of microbial community alpha diversity and the level of cytokines in blood serum. Correlations were considered between alpha diversity indices and cytokines that had previously been identified as significant. The presence of a relationship was demonstrated only for the macrophage-derived chemokine MDC (Figure 6).

Based on Figure 6, the MDC chemokine level has a negative correlation (R) with the Shannon, Pielou, and Simpson indices and only a positive one with the Strong index. The level of significance of all correlations is reliably significant (α = 0.05); accordingly, even a small coefficient should be taken into account.

**Figure 7:**
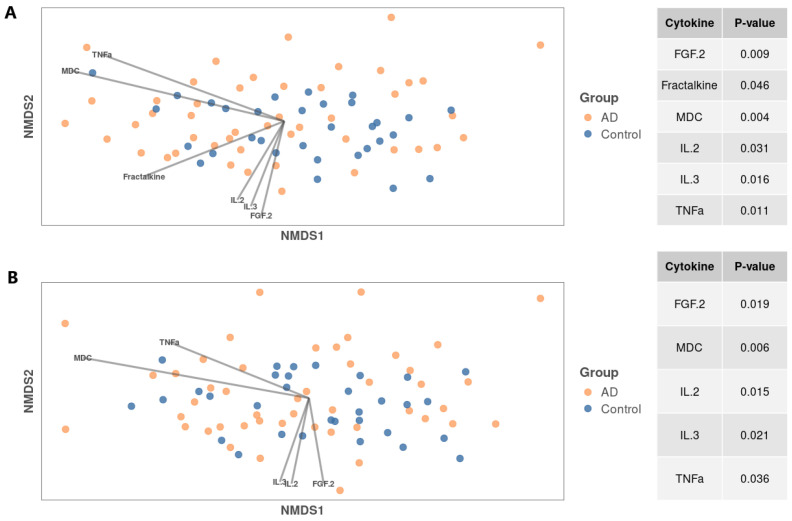
NMDS analysis results for RDP (**A**) and SILVA (**B**) in a joint analysis of the two groups; on the right side of the figure is a table where the first column contains the names of statistically significant cytokines, and the second column contains adjusted *p*-values.

To identify cytokines that affect the structure of the bacterial community, we conducted a multidimensional analysis (Figure 7).

**Figure 8:**
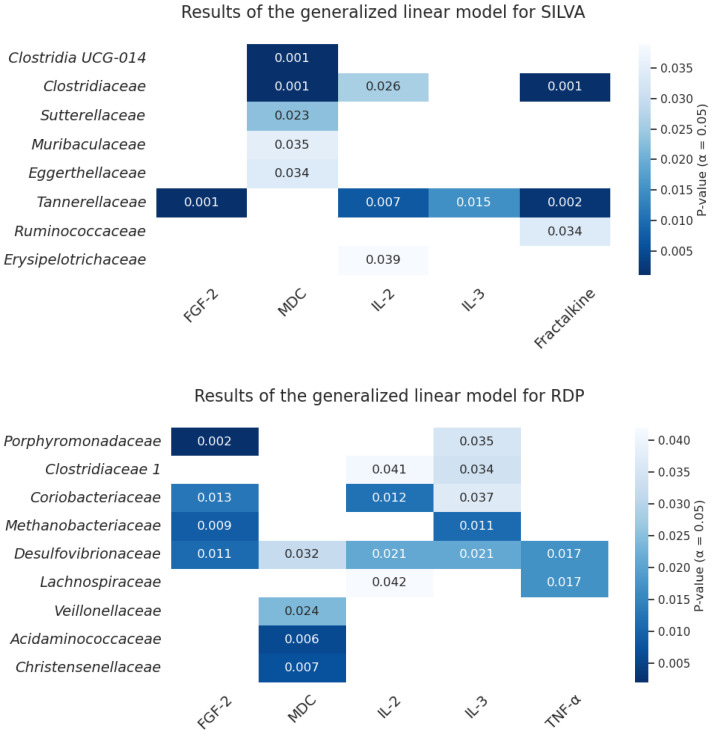
Results of the generalized linear model for RDP and SILVA.

Six significant cytokines were identified that covaried with the structure of the bacterial community, potentially causing changes in it when analyzing the RDP data (fibroblast growth factor 2 (FGF-2), fractalkine, MDC, IL-2, IL-3, and TNF-α), and five cytokines/chemokines for the SILVA data (same list with the exception of fractalkine). Next, a generalized linear model (glm) was used to determine which cytokine families in the atopic dermatitis group were associated with significant results (Figure 8).

### 3.5. Correlations between Individual Bacterial Taxa and Cytokines/Chemokines

**Figure 9:**
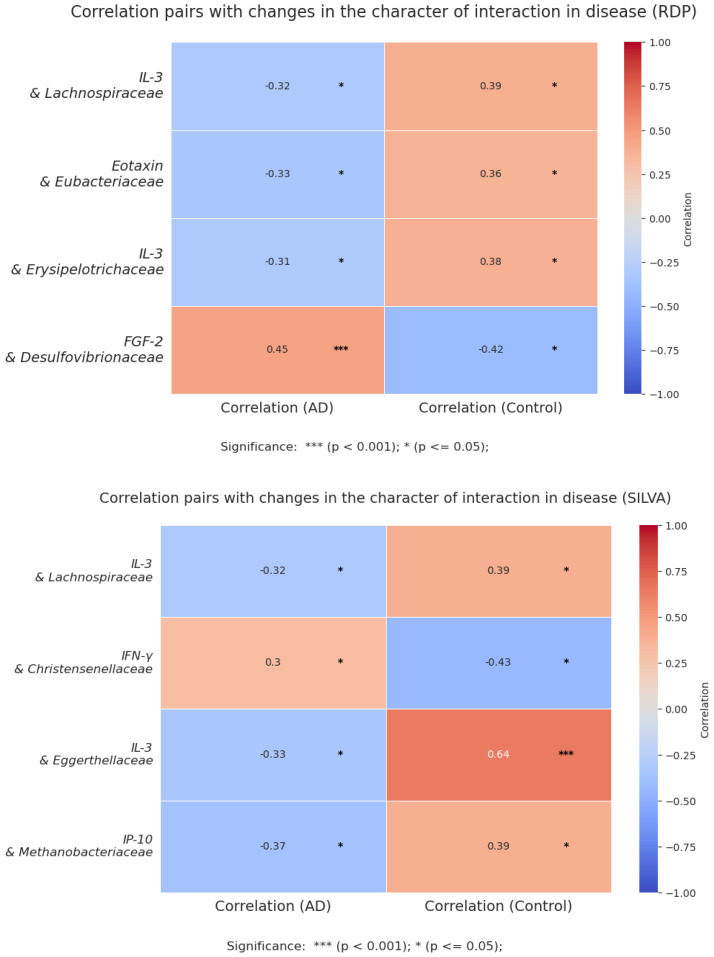
Correlation pairs with changes in the character of interaction in disease (RDP and SILVA).

A correlation analysis was performed between the level of cytokines and the relative number of bacteria within each group. Spearman’s method was used for the calculation. Based on the results obtained, many relationships undergo changes in AD. From all the results obtained, pairs corresponding to two conditions were selected: (1) statistical significance was confirmed in the two studied groups (AD and control) and (2) the correlation coefficient of the pair showed values opposite in sign between the groups, indicating a change in the character of interaction (Figure 9).

For a more detailed analysis of the correlations between circulating cytokine/chemokine concentrations and individual bacterial taxa in the intestinal microbiota of children with atopic dermatitis, heat maps were created and are presented in Figure S3. During the visualization, bacteria and cytokines that had no statistically significant correlation were removed from the graph to reduce noise.

## 4. Discussion

In this study, we characterized the structure and composition of the gut microbiota, as well as the cytokine/chemokine profile, in children. In a cohort of children with AD, a change in the overall structure of the microbiome was demonstrated relative to a group of healthy children. In particular, we observed changes in alpha (according to the Shannon, Pielou, Simpson, and Strong indices) and beta diversity. LEfSe and the coda4microbiome algorithm identified several potentially significant bacterial groups for the diagnosis and/or therapy of atopy, such as *Pasteurellaceae*, *Barnesiellaceae*, *Eubacteriaceae*, *Clostridiales Incertae Sedis XIII*, *Oscillospiraceae*, *Peptococcaceae*, *Anaerovoraceae*, and *Flavobacteriaceae*. In addition, participants with AD showed changes in the profiles of circulating cytokines and chemokines, namely, increased levels of MDC/CCL22, IL-1ra, IL-12p70, IL-5, IL-8, IL-13, MIP-1α/CCL3, IFN-α2, Flt3L (fms-related tyrosine kinase 3 ligand), G-CSF (granulocyte colony-stimulating factor), TGF-α (transforming growth factor alpha), IFN-γ, TNF-α, and VEGF and reduced levels of IL-2, IL-1α, IL-15, and IL-17A. A pronounced correlation was also found between the differential cytokine/chemokine content and changes in the gut microbiota in children with atopic dermatitis. This indicates that intestinal dysbiosis is accompanied by a complex proinflammatory reaction in pediatric patients with AD, which may play an important role in the pathogenesis of atopy.

Atopic dermatitis is a systemic disease that often precedes the occurrence of other inflammatory conditions such as asthma, allergic rhinitis, or food allergies [5]. The mechanism of pathogenesis of atopy has not been fully studied; however, it has been shown that the most significant role is played by the dominance of the Th2-type immune response in the acute phase [7,34] and Th17 in the chronic phase [35]. Since a possible cause of atopic inflammation could be the dysbiosis of the intestine and the dysfunction of the intestinal barrier [36,37], it is necessary to identify the different bacterial groups in patients with atopy compared to a control group. The analysis showed that patients had a significant increase in the abundance of certain bacterial groups such as *Pasteurellaceae*, *Peptococcaceae*, and *Clostridia* UCG-014, while there was a decrease in other groups such as *Barnesiellaceae*, *Eubacteriaceae*, *Clostridiales Incertae Sedis XIII*, *Oscillospiraceae*, *Anaerovoraceae*, and *Flavobacteriaceae* compared to the control group. We also noted a decrease in alpha diversity in patient samples, which is consistent with an early study where children with less microbiota diversity were more susceptible to atopic dermatitis [38]. These results suggest that a change in the gut microbiota may be associated with the development of systemic inflammation. In our previous study [27], we also observed an increase in the abundance of members of the *Pasteurellaceae* family and a decrease in members of the *Barnesiellaceae* family in individuals with an atopic phenotype. However, in this study, we aimed to investigate the relationship between changes in the intestinal microbiota and immune responses in the pathogenesis of atopic diseases. The *Pasteurellaceae* family is an important bacterial signature in immune system disorders [39]. For example, it was previously associated with a proinflammatory phenotype in obesity [40]. In addition, studies have noted an increase in the abundance of this group in the gut microbiota of individuals with AD [37,41], as well as an association with allergic inflammation [42]. It is known that variability in the composition of the gut microbiota can be influenced by various factors, such as the region of residence, season, genetics, infectious diseases, hematological conditions, metabolic disorders, and autoimmune diseases. To minimize the influence of these factors in our study, we tried to control for them as much as possible. Despite this, we acknowledge that the microbiota composition at a given point in time may not always accurately reflect the metabolic activity of the bacteria. However, our data show a consistent pattern with previous studies [5,25,26], suggesting that the microbiota of children with atopic dermatitis in our work has less diversity and a greater proinflammatory potential. This suggests a possible role for the “gut–skin axis” in the development of atopic conditions [12].

After identifying differences in the bacterial signatures of the gut microbiota between patients, we attempted to characterize the relationship between these differences and systemic inflammation. Cytokine/chemokine levels in serum reflect the current state of the immune system, so we conducted a comparative analysis to determine the degree of polarization of the immune response. A sufficient number of biomarkers for atopic dermatitis have been described in the literature [8]. However, there is heterogeneity among AD endotypes, indicating the need for different biomarkers in specific subpopulations [43,44]. A study using multiplex analysis revealed a statistically significant increase (*p* < 0.05) in the quantitative levels of several cytokines and chemokines that are characteristic of atopic conditions (Figure 5). These include MDC/CCL22, MIP-1α/CCL3, IL-5, IL-8, IL-13, IFN-γ, TNF-α, and VEGF. This suggests that there may be a systemic inflammatory response in these patients. MDC/CCL22 (macrophage-derived chemokine) is a Th2-associated chemokine and belongs to the key mediators of atopic disorders [8,45]. It is remarkable that in our study, correlations between MDC/CCL22 and bacterial taxa associated with atopic dermatitis were revealed. A positive relationship was found with the *Pasteurellaceae* family, while a negative correlation was observed with such important groups as *Barnesiellaceae*, *Oscillospiraceae*, *Peptococcaceae*, *Eubacterium coprostanoligenes*, and *Clostridia UCG-014*. Furthermore, the level of the chemokine correlated with α-diversity indices, indicating a close relationship between MDC and the intestinal microbiome in the pathogenesis of atopic dermatitis. As our study has shown, changes in the concentration of the macrophage-derived chemokine are associated with alterations in the composition of the bacterial community. Specifically, an increase in chemokine concentration is linked to a decrease in richness and diversity (as measured by the Shannon, Pielou, and Simpson indices) and an increase in relative inequality among the members of the microbiota (as indicated by the Strong index). Additionally, there is a decrease in the abundance of certain bacterial taxa in the gut (as shown in Figure 8 and Figure S3), which is also correlated with changes in chemokine levels. These observations emphasize the significant role of the *Pasteurellaceae* bacterial family and the MDC/CCL22 chemokine in the development of AD, which requires the study of more detailed pathogenesis mechanisms.

Despite the fact that the number of *Clostridia UCG-014* is increased in participants with AD, this group of bacteria is considered to be a useful component of the intestinal microbiota [46]. This may be indicated by a negative correlation with inflammatory markers such as MDC/CCL22 and TNF-α, which have also been shown to be elevated in this group. A negative correlation of this family with TNF-α was also previously observed in mice with ulcerative colitis [47], and an increase in *Clostridia UCG-014* levels was noted in patients with persistent atopic dermatitis in a Chinese study [48], suggesting an important role for this family in systemic inflammation.

The results of our study are consistent with previous research. An increase in TNF-α levels is noted in the blood of patients with atopy and is reported to correlate with the severity of the disease, since it plays an important role in inflammatory reactions [49]. TNF-α has been linked to Th1/Th17 responses [50], which may indicate a chronic course of the disease [11], as well as the need for further study of the molecular mechanisms of the pathogenesis of AD and the construction of cytokine networks characteristic of this pathology. Our data also suggest a possible link between TNF-α and changes in the number of specific bacteria in the colonic mucosa (Figure 8), which may contribute to pathological inflammation, such as *Desulfovibrionaceae* and *Lachnospiraceae* [51,52]. Thus, it has been previously noted that commensal bacteria can cause damage to the intestinal barrier, altering the production of proinflammatory cytokines by the intestinal epithelium, such as IL-8 and TNF-α [53]. However, it is important to note the positive relationship of *Desulfovibrionaceae* with IL-1ra and FGF-2, as well as the negative relationship with MIP-1β. *Lachnospiraceae* correlates negatively with the proinflammatory factor IL-3 and changes the nature of interaction in the control group. These data indicate an ambiguous role of these bacterial families in inflammation development. This provides a basis for studying the role of bacteria in the pathogenesis of atopy.

Bacteria that produce short-chain fatty acids (SCFAs) are important to note, as the dysbiosis and imbalance associated with these taxa can lead to systemic immune changes [15]. These SCFAs play a significant role in immune regulation, as they help regulate the functions of various types of immune cells and participate in different stages of the inflammatory response. They also help regulate the production of certain types of immune cells, such as Th1, Th17, and Treg cells, and can inhibit the production of proinflammatory cytokines [54]. In our study, we observed a decrease in the numbers of certain groups of bacteria that produce SCFAs, such as *Eubacteriaceae* and *Clostridiales*, in the group with AD. This could contribute to increased concentrations of proinflammatory cytokines and lead to immune dysfunction. The *Eubacteriaceae* family is an essential part of the healthy gut microbiota and produces butyrate, which has been linked to several health benefits [55]. Our findings suggest that a decrease in this family’s abundance in children with AD may contribute to their immune imbalance. In addition, with an increase in serum biomarkers of inflammation, such as eotaxin/CCL11, MDC/CCL22, and Flt3L, there is a decrease in the content of *Eubacteriaceae*. There is also a change in the interaction between the important atopy marker eotaxin/CCL11 and the number of this bacterial family (Figure 9). This may indicate an association between dysbiosis and atopic disorders. A decrease in this bacterial family has also been noted in other inflammatory conditions previously [56]. However, detailed mechanisms for their influence on immunity need to be studied further. A negative correlation of *Clostridiaceae* with IL-2, IL-3, and vascular endothelial growth factor (VEGF) was found. Previously, it has been shown that members of the *Clostridiaceae* family can effectively influence immune responses and specifically induce the production of Th17 cell cytokines in mice [57]. This suggests that not only metabolites but also bacterial antigens may be involved in immune reactions [35]. A decrease in the number of representatives of the *Barnesiellaceae* and *Oscillospiraceae* families was also shown in children with AD. A decrease in the number of *Oscillospiriaceae* members in the microbiota of children with acute atopic dermatitis was also observed in another Russian study. These bacteria can have a positive effect on the mucous membrane of the gastrointestinal tract thanks to their ability to produce butyrate and help prevent the development of inflammation [58]. Other studies have previously noted a decrease in representatives of the *Barnesiellaceae* family in IgE-mediated food allergies [59], as well as in other inflammatory diseases [60]. We noted the negative relationship of this family with such Th2 signature cytokines as IL-5 and IL-9, which confirms the potentially anti-inflammatory role of these families.

There is insufficient data on the involvement of *Clostridiales Incertae Sedis XIII*, *Anaerovoracaceae*, and *Flavobacteriaceae* in inflammation, including atopy; however, a decrease in the relative number of representatives *of Clostridiales Incertae Sedis XIII* and *Flavobacteriaceae* was observed in a Chinese study of IgE-mediated food allergy in infants [61]. A negative correlation with such serum markers of inflammation as MDC/CCL22 and TNF-α suggests that these groups of bacteria may have immunomodulatory properties. It was previously noted that *Clostridiales Incertae Sedis XIII* strains obtained from the human gut microbiome can promote the induction of Treg cells in the colon and inhibit the development of a Th2 response in models of colitis and allergic diarrhea in mice [62]. These data may indicate that the interaction of the intestinal microbiota with T cells and intestinal B cells may lead to a systemic effect on the immune system.

Vascular endothelial growth factor (VEGF) is considered as a factor stimulating angiogenesis and vascular permeability and is probably associated with an inflammatory response [63]. In some studies, there is an increased secretion of VEGF in atopy [64,65]. The bacterial families *Methanobacteriaceae* and *Coriobacteriaceae* negatively correlated with the concentration of this marker in our study in a group of participants with atopic dermatitis. The archaea family *Methanobacteriaceae* is a representative of the normal human gut microbiome and an important participant in metabolic pathways in the gastrointestinal tract, and also positively correlated with BMI (body mass index) in another study [66]. The relationship of this family with atopic disorders has not been previously observed; however, an increase in the number of representatives of this family is associated with IBD (inflammatory bowel disease), irritable bowel syndrome, and obesity [67,68]. *Methanobacteriaceae* plays an important role in the intestinal ecosystem, contributing to the fermentation of carbohydrates and the production of SCFAs by commensal bacteria [69]. *Coriobacteriaceae* is also representative of the normal microbiota of the gastrointestinal tract and performs important functions such as the conversion of bile acid salts and steroids, as well as the activation of food polyphenols [70]. Due to the fact that these families are involved in important metabolic pathways, it can be assumed that dysbiosis of these families may be associated with various immune disorders, including atopy. In our work, we also noted a negative relationship with inflammatory mediators such as fibroblast growth factor-2 (FGF-2), IL-**1**β, IL-1ra, and IL-2, as well as another important biomarker of atopy—MIP-1α/CCL3. Curiously, we were able to note a change in the nature of the interaction between *Methanobacteriaceae* and IP-10 in normal and pathological conditions, which also suggests that this family is associated with intestinal health. However, *Methanobacteriaceae* was negatively correlated with the anti-inflammatory marker IL-1ra, which is in contrast to the findings.

VEGF levels in our study were positively correlated with potentially proinflammatory bacterial families such as *Desulfovibrionaceae* and *Acidaminococcaceae.* Presumably, representatives of these families are able to induce the production of VEGF, while VEGF production is also enhanced by subsequent hyperproduction of IL-13 [71]. In addition, we noted a positive correlation of these families with FGF-2 and IL-3, which indicates the complex nature of the relationship between the gut microbiota and systemic immunity.

Another important bacterial marker of intestinal health is the *Christensenellaceae* family. We observed a negative correlation with signaling molecules such as MDC/CCL22 and TNF-α in atopy. It is important to note that *Christensenellaceae* and IFN-γ change the nature of interaction in the case of the disease: a positive correlation is observed in AD, and a negative one is normal. The positive relationship between this family and transforming growth factor alpha (TGF-α) also warrants attention. These observations highlight the significant role of this family in human health and its involvement in immune processes.

We observed a negative correlation between the concentration of another important atopy-related chemokine, eotaxin-1/CCL11, and the abundance of members of the *Eubacteriaceae* and *Muribaculaceae* families in AD. In addition, we observed a positive correlation between this cytokine and *Erysipelotrichaceae* and *Pasteurellaceae* families, which, as noted previously, have proinflammatory potential and are associated with atopic disorders [27,39]. Eotaxin-1/CCL11 is an important biomarker of atopic dermatitis involved in the selective recruitment of eosinophils into inflammatory foci [72,73], thereby being an important participant in allergic and atopic reactions. However, *Muribaculaceae* has a negative correlation with the anti-inflammatory cytokine IL-10, while *Erysipelotrichaceae* has a negative correlation with the proinflammatory cytokines IL-1β and IL-2. This indicates some contradictions in our data.

The role of TGF-α and G-CSF in atopic inflammation has not been described in detail, although it is assumed that they are important for wound healing in response to allergen exposure [8,74,75]. In our study, there was an increase in the concentration of these growth factors in the group with AD relative to the control, as well as a negative correlation with the *Erysipelotrichaceae* family.

Overall, the effects and patterns we observed could be caused by the fact that if the balance of the bacterial ecosystem of the intestine was disrupted, an excessive growth of potentially pathogenic groups of bacteria could occur, and a decrease in the number of beneficial bacteria could lead to an increase in the permeability of the intestinal barrier. A decrease in the protective function of the intestinal barrier could initiate a systemic modulation of the organism’s immune processes due to the entering of the bloodstream of food allergens, bacterial metabolites, bacterial antigens or endotoxins, and other agents that can cause excessive production of proinflammatory cytokines and chemokines [8], as well as activation of Th2 cells [53]. This could potentially lead to excessive production of Th2- and Th2-related signaling molecules, activation of B cells and the production of IgE, eosinophilia, and the release of downstream inflammatory mediators and the induction of clinical symptoms of atopic dermatitis [43]. These effects could be suppressed by anti-inflammatory metabolites produced by certain groups of intestinal microorganisms. In addition, the intestinal microbiota is able to influence the immune system through the signaling pathways of toll-like receptors; therefore, a decrease in the relative number of potentially immunomodulating intestinal bacteria could be associated with increased cytokine reactions to TLR ligands and the development of atopy [35,76]. Collectively, it can be assumed that intestinal dysbiosis in various ways could lead to immune dysregulation and the development of an atopic condition.

Despite the fact that AD shows a polarization of the immune response toward Th2 cells, it is important to consider that the immune system of children is still developing under the influence of various environmental factors. These factors can contribute to an increase in the levels of Th1-related cytokines, which are important for the immune system’s defense against pathogens. Furthermore, during the development of atopic conditions such as AD, an increase in the number of polarized Th2 cells can lead to an increase in both Th1 and regulatory cytokine levels through a feedback mechanism [77]. Additionally, it is important to consider the gender and age-related characteristics of the immune response when studying atopic conditions in children [78]. These factors could explain the increased levels of cytokines observed in our study that are not typically associated with atopy, as well as the mixed effects seen in terms of the increased levels of Th1-response cytokines such as IFN-γ and IL-12p70. For example, we observed that in the group of children with AD, the levels of the proinflammatory cytokine IL-17A were lower compared to the control group. We hypothesize that this pattern may be due to the fact that IL-4 has the potential to inhibit the production of IL-17A [79].

The correlation between serum biomarkers that are important for atopy and certain bacterial taxa that are involved in dysbiosis in this pathology suggests the significant role of the intestinal microbiota in modulating immune responses and the potential for correcting the intestinal microbiota as part of the treatment for these conditions. Therefore, further research into this relationship is necessary. Thus, the complex interactions between cytokines, chemokines, and the gut microbiota in the development of AD require further investigation.

This study has some limitations, and the main one was the small number of participants and samples. Due to difficulties in working with children, we were able to collect only one blood and one stool sample from each participant. Although this was the largest possible group of participants according to the decision of the ethics committee, it should be taken into consideration in future studies.

Another limiting factor was the timeframe of the study. Because the study was con-ducted during cold and cloudy months, it was not possible to account for the impact of seasonal changes on the composition of the participants’ intestinal microbiota or cytokine profile, as well as the effects of vitamin D.

The next important limitation is the difficulty in diagnosing atopic dermatitis, as well as the personal variability in cytokine profiles. This disease does not have a specific laboratory test, so we attempted to create a group of participants with a reliably established diagnosis according to IgE levels and the Rajka criteria (HRC) [80]. There is also a limited amount of research available in this area to compare our work with.

Additionally, it is difficult to determine whether the level of cytokines in the gut affects the composition of the intestinal bacteria or intestinal dysbiosis initiates changes in the content of inflammation markers in blood serum; further research will help to answer this question. The understanding of these complex interactions could have a significant impact on the development of personalized medicine and improved patient outcomes.

## 5. Conclusions

The microbiome of pediatric patients suffering from atopic dermatitis exhibited notable signs of deviation from the healthy microbial community structure. This dysbiosis is hypothesized to contribute to intestinal barrier function loss, thereby increasing its permeability. Such alterations in the gut barrier facilitate translocation of microbial products or antigens into the systemic circulation, thus altering the balance of the immune system.

Such dysbiotic conditions shift the Th1/Th2 cell ratio, which is a critical factor in the pathogenesis of allergic diseases, including atopic dermatitis.

Alterations in gut microbiome relative abundance appeared to correlate with shifted levels of cytokines. Thus, the obtained results added to the evidence of the gut microbiota’s influence extending beyond local gut inflammation to encompass systemic inflammatory processes. Also, changes in the relative abundance of gut microbiome representatives showed a correlation with the serum levels of both proinflammatory and anti-inflammatory cytokines, indicating a potential mechanistic link between gut bacteria and the immune responses observed in atopic dermatitis patients.

Our findings suggest that specific microbial profiles in the gut might have conferred an increased risk for the development of atopic dermatitis in children and could have modulated immune responses.

## Figures and Tables

**Table 1:** Results of beta diversity analysis. PERMANOVA results for UniFrac ordination data.

		F-Value	*p*-Value
Unweighted	RDP	5.1	0.026
	SILVA	5.4	0.021
Weighted	RDP	3.7	0.06
	SILVA	5.4	0.03

## Data Availability

The datasets used and analyzed in the present study are available from the corresponding author on reasonable request.

## Supplementary Materials

The following supporting information can be downloaded at https://www.mdpi.com/article/10.3390/biomedicines12102351/s1, Table S1: Description of cytokines and chemokines analyzed in the study; Figure S1: The results of the beta diversity analysis. Ordinal analysis of UniFrac in 3-dimensional visualization: (A,B)—weighted and unweighted analysis of UniFrac for RDP data (respectively); (C,D)—weighted and unweighted analysis of UniFrac for SILVA data (respectively), Figure S2: LEfSe results for RDP (A) and SILVA (B) data: cladograms of bacterial families with significant differences in groups, Figure S3: Correlation analysis of serum biomarker profiles with individual bacterial taxa for RDP (A) and SILVA (B) data, respectively, for the group with atopic dermatitis (AD). Red represents a positive correlation, and blue represents a negative correlation. * *p* < 0.05; ** *p* < 0.01; *** *p* < 0.001.
