# Supplementary material for: The Association between Gut Microbiota and Serum Biomarkers in Children with Atopic Dermatitis
Source: Biomedicines. 2024 Oct 15;12(10):2351. doi: 10.3390/biomedicines12102351 (PMC11505256; doi:10.3390/biomedicines12102351)
Supplement: Supplementary file 1 [file biomedicines-12-02351-s001.zip › biomedicines-3197199-supplementary.pdf]

Supplementary materials:

Table S1. Description of cytokines and chemokines analyzed in the study.

Abbreviation	Description	Family
**sCD40L**	The soluble form of the CD40 ligand	TNF superfamily
**EGF**	Epidermal Growth Factor	Growth Factor
**Eotaxin-1/CCL11**	C-C motif chemokine 11, eosinophil chemotactic protein	Chemokine
**FGF-2**	Fibroblast growth factor 2	Growth Factor
**Flt-3L**	Fms-related tyrosine kinase 3 ligand	Growth Factor
**Fractalkine /CX3CL1**	Chemokine (C-X3-C motif) ligand 1	Chemokine
**G-CSF**	Granulocyte colony-stimulating factor	Growth Factor
**GM-CSF**	Granulocyte-macrophage colony-stimulating factor	Growth Factor
**GROα/CXCL1**	Growth regulated oncogene-alpha/The chemokine (C-X-C motif) ligand 1	Chemokine
**IFN-α2**	Interferon alpha-2	Interferons
**IFN-γ**	Interferon gamma	Interferons
**IL-1α**	Interleukin-1 alpha/ hematopoietin 1	Interleukin 1
**IL-1β**	Interleukin-1 beta/ leukocytic pyrogen	Interleukin 1
**IL-1ra**	The interleukin-1 receptor antagonist	Interleukin 1
**IL-2**	Interleukin-2	Interleukin
**IL-3**	Interleukin-3	Interleukin
**IL-4**	Interleukin-4	Interleukin
**IL-5**	Interleukin-5	Interleukin
**IL-6**	Interleukin-6	Interleukin
**IL-7**	Interleukin-7	Interleukin
**IL-8**	Interleukin-8	Interleukin
**IL-9**	Interleukin-9	Interleukin
**IL-10**	Interleukin-10	Interleukin
**IL-12 (p40)**	Subunit beta of interleukin 12/ interleukin-12 subunit p40	Interleukin
**IL-12 (p70)**	Interleukin 12 subunit p70	Interleukin
**IL-13**	Interleukin-13	Interleukin
**IL-15**	Interleukin-15	Interleukin
**IL-17A**	Interleukin-17A	Interleukin
**IP-10/CXCL10**	C-X-C motif chemokine ligand 10/ Interferon gamma-induced protein 10	Chemokine
**MCP-1/CCL2**	The chemokine (C-C motif) ligand 2/ monocyte chemoattractant protein 1	Chemokine
**MCP-3/ CCL7**	Chemokine (C-C motif) ligand 7/ monocyte-chemotactic protein 3	Chemokine
**MDC/CCL22**	C-C motif chemokine 22/ Macrophage-Derived Chemokine	Chemokine
**MIP-1α/CCL3**	Chemokine (C-C motif) ligand 3/ Macrophage Inflammatory Protein 1-α	Chemokine
**MIP-1β/CCL4**	Chemokine (C-C motif) ligand 4/ Macrophage Inflammatory Protein 1-β	Chemokine
**PDGF-AA**	Platelet-derived growth factor	Growth factors
**PDGF-AB/BB**	Platelet-derived growth factor	Growth factors
**RANTES/ CCL5**	Chemokine (C-C motif) ligand 5/ Regulated on Activation, Normal T-cell Expressed and Secreted	Chemokine
**TGF-α**	Transforming growth factor alpha	Growth factors
**TNF-α**	Tumor necrosis factor alpha	TNF superfamily
**TNFβ/ LT-α**	Lymphotoxin-alpha/ tumor necrosis factor-beta	TNF superfamily
**VEGF**	Vascular endothelial growth factor	Growth factors

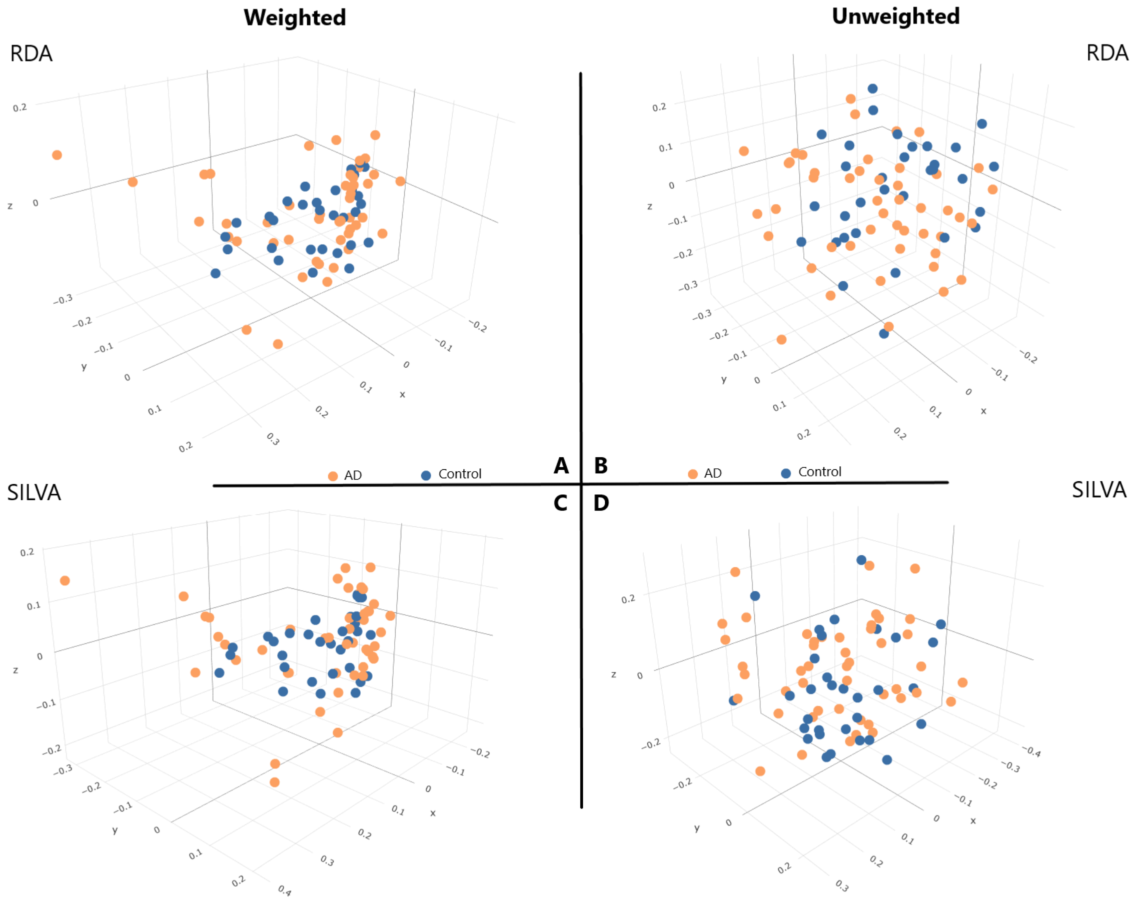

Figure S1. The results of the beta diversity analysis. Ordinal analysis of UniFrac in 3-dimensional visualization; A, B – weighted and unweighted analysis of UniFrac for RDP data (respectively), C, D – weighted and unweighted analysis of UniFrac for SILVA data (respectively).

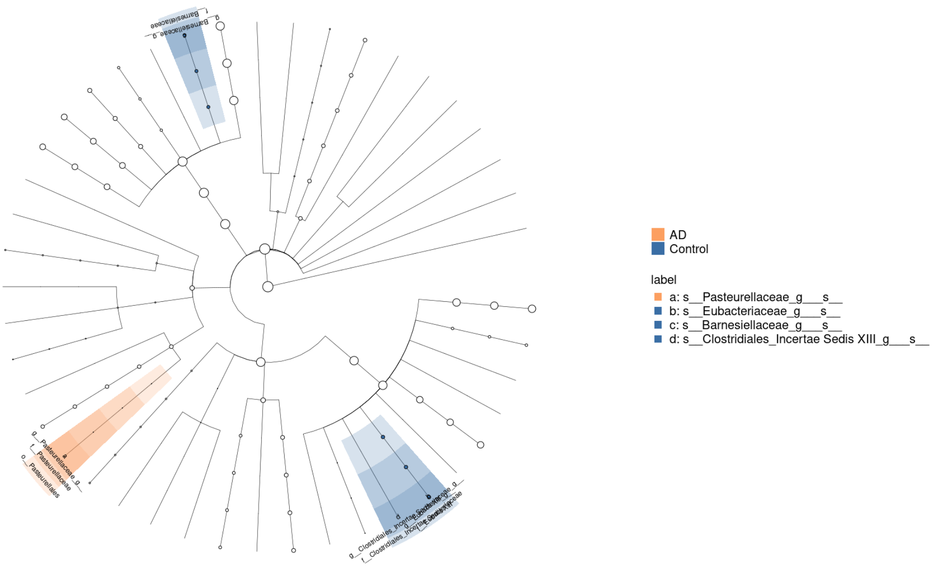

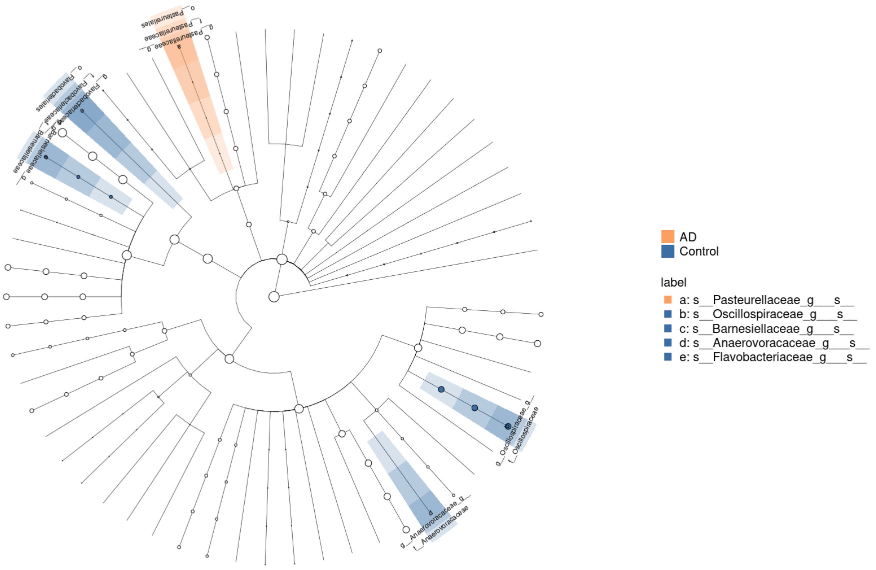

Figure S2. LEfSe results for RDP (A) and SILVA(B) data; cladograms with bacterial families with significant differences in groups.

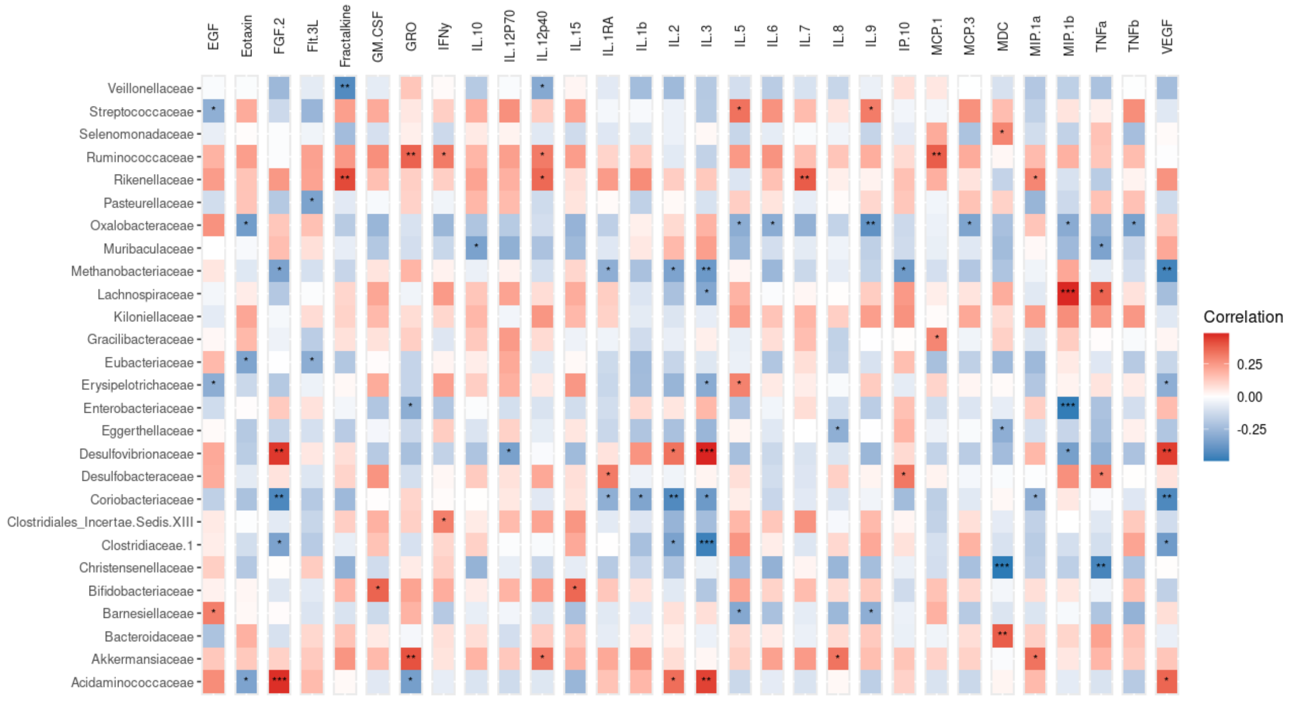

A

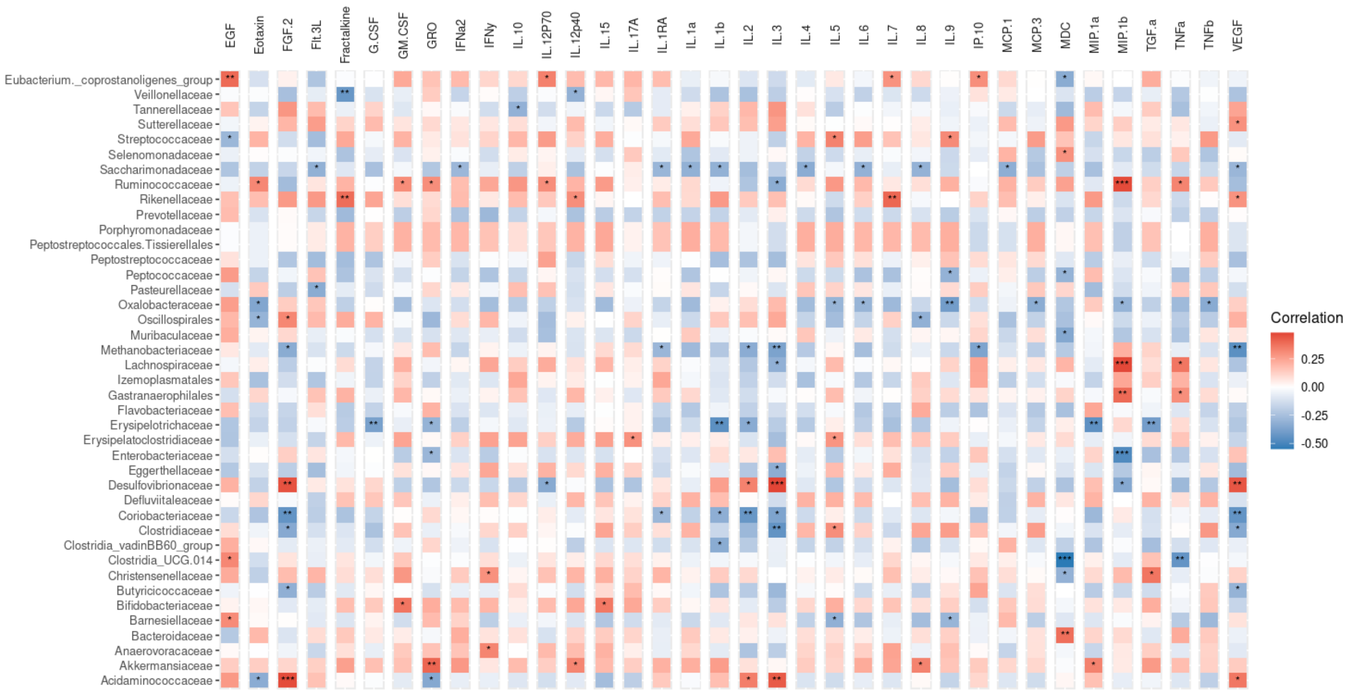

B

Figure S3. Correlation analysis of serum biomarker profiles with individual bacterial taxa for RDP (A) and SILVA (B) data, respectively, for the group with atopic dermatitis (AD). Red represents positive correlations, and blue represents negative correlations; *p<0.05; **p<0.01; ***p<0.001.

Patient informed consent

I, ____,

confirm, *(Full name)*

that I have received complete information regarding the activities of the Federal State Budgetary Institution «Centre for Strategic Planning and Management of Biomedical Health Risks» of the Federal medical and biological agency regarding the procedure for collecting, storing and analyzing my biosamples. My participation in research is voluntary. I have signed an agreement on the processing of personal data in accordance with the legislative acts of the Russian Federation. I reserve the right to refuse or terminate my participation in research at any time without giving any reason. In this case, I will not be subject to any infringement of my rights or loss of any advantage that belongs to me. I am informed that during the blood collection procedure, minor reactions of the body are possible (short-term decrease in blood pressure, hematoma in the puncture area), which are not the result of personnel error.

I agree to undergo the procedure of taking a biosample of intestinal microflora and blood samples for laboratory tests.

I agree for the long-term storage of my personal biosamples of symbiotic microflora and blood samples for scientific use.

I agree for analyze my biosamples and transfer anonymized research results to third parties in agreement with the Centre for Strategic Planning, of the Federal medical and biological agency, in the event that this does not infringe on my rights and does not lead to the loss of any advantage belonging to me. I am informed that I can receive all the analysis results of my biospecimens, but do not have the right to transfer them to third parties.

I agree that my personalized medical data collected by the physician-researcher was reviewed by authorized persons responsible for maintaining medical confidentiality and ethics in conducting scientific research, assessing research results and preparing publications in the scientific literature. In this case, information about my health will be confidential, and my personal data will be used in an impersonal form.

I agree to allow specialists access to all medical records and data, as well as discharge documents and post-mortem examination data in the event of my death.

___ __________ 20___ ________________/________________

date *donor signature / donor's full name*

___ __________ 20___ ________________/________________

date *employee's signature / employee's full name*

___ __________ 20___ ________________/________________

date *employee's signature / employee's full name*
